# Species delimitation by DNA barcoding reveals undescribed diversity in Stelliferinae (Sciaenidae)

**DOI:** 10.1371/journal.pone.0296335

**Published:** 2023-12-28

**Authors:** Tárcia Fernanda da Silva, Iracilda Sampaio, Arturo Angulo, Omar Domínguez-Domínguez, Jonas Andrade-Santos, Aurycéia Guimarães-Costa, Simoni Santos

**Affiliations:** 1 Laboratory of Fish Microbiology, Institute of Coastal Studies, Federal University of Para (UFPA), Braganca, Para, Brazil; 2 Laboratory of Evolution, Institute of Coastal Studies, Federal University of Para (UFPA), Braganca, Para, Brazil; 3 Escuela de Biología, Museo de Zoología/Centro de Investigación en Biodiversidad y Ecología Tropical (CIBET) and Centro de Investigación en Ciencias del Mar y Limnología (CIMAR), Universidad de Costa Rica, San Pedro de Montes de Oca, San José, Costa Rica; 4 Laboratory of Aquatic Biology, Faculty of Biology, Universidad Michoacana de San Nicolás de Hidalgo (UMSNH), Morella, Michoacán, Mexico; 5 Laboratory of Ichthyology, Vertebrates Department–Federal University of Rio de Janeiro, National Museum, Rio de Janeiro, Brazil; Pontificia Universidade Catolica do Rio Grande do Sul, BRAZIL

## Abstract

Stelliferinae is the third most speciose subfamily of Sciaenidae, with 51 recognized species arranged in five genera. Phylogenies derived from both morphological and molecular data support the monophyly of this subfamily, although there is no general consensus on the intergeneric relationships or the species diversity of this group. We used the barcoding region of the cytochrome oxidase C subunit I (COI) gene to verify the delimitation of Stelliferinae species based on the Automatic Barcode Gap Discovery (ABGD), Generalized Mixed Yule Coalescence (GMYC), and Bayesian Poisson Tree Process (bPTP) methods. In general, the results of these different approaches were congruent, delimiting 30–32 molecular operational taxonomic units (MOTUs), most of which coincided with valid species. Specimens of *Stellifer menezesi* and *Stellifer gomezi* were attributed to a single species, which disagrees with the most recent review of this genus. The evidence also indicated that *Odontoscion xanthops* and *Corvula macrops* belong to a single MOTU. In contrast, evidence also indicates presence of distinct lineages in both *Odontoscion dentex* and *Bairdiella chrysoura*. Such results are compatible with the existence of cryptic species, which is supported by the genetic divergence and haplotype genealogy. Therefore, the results of the present study indicate the existence of undescribed diversity in the Stelliferinae, which reinforces the need for an ample taxonomic review of the fish in this subfamily.

## Introduction

Sciaenidae is a family of fish classified in the order Acanthuriformes [1], which is widely distributed in the tropical, subtropical, and temperate waters of the Atlantic, Indian, and Pacific oceans, as well as in rivers of both the Old and New Worlds [2, 3]. Sciaenidae is a large family, with 298 species in 68 genera [3, 4]. Phylogenetic inferences, based on both morphological and molecular data, have confirmed the monophyly of the Sciaenidae [5–7], and the multilocus phylogeny of Lo et al. [7] delimited 15 well-supported lineages, including the subfamily Stelliferinae, which is composed of six genera–*Stellifer* Oken, 1817, *Ophioscion* Gill, 1863, *Bairdiella* Gill, 1861, *Odontoscion* Gill, 1862, *Corvula* Jordan and Eigenmann, 1889, and *Elattarchus* Jordan and Evermann, 1896.

Stelliferinae is the third most speciose sciaenid subfamily, with 51 valid species [3–5, 8]. The stelliferine species has an amphi-American distribution, occurring in coastal and estuarine environments in the western Atlantic and eastern Pacific [5, 8]. Although there is a consensus on the monophyly of the subfamily [5, 7, 9, 10], the phylogenetic relationships among the different genera are still not completely resolved. In fact, the taxonomy of the group is still undergoing review, given that many clades are poorly defined due to the overlap in morphological traits and their genetic similarity, which is probably the result of the explosive adaptive radiation of the group [9, 10].

Phylogenetic analyses based on multilocus data have shown that *Stellifer* and *Ophioscion* are not monophyletic, as suggested previously, and thus require a detailed taxonomic review [7, 9, 10]. More recently, Chao et al. [8] recognized *Ophioscion* as a junior synonym of *Stellifer* based on morphological analysis, which means that Stelliferinae is currently made up of only five genera (*Stellifer*, *Bairdiella*, *Odontoscion*, *Corvula*, and *Elattarchus*). Chao et al. [8] also described five new *Stellifer* species–*Stellifer cervigoni*, *Stellifer collettei*, *Stellifer musicki*, *Stellifer macallisteri*, and *Stellifer menezesi*, the latter previously identified as lineage II of *Ophioscion punctatissimus* Meek and Hildebrand, 1925 by Silva et al. [10]. Additionally, Marceniuk et al. [11] revised the taxonomy of *Bairdiella* from the western South Atlantic based on both morphology and DNA barcoding and concluded that *Bairdiella ronchus* Cuvier, 1830 is in fact a species complex that includes *B*. *ronchus*, the revalidated *Bairdiella veraecrucis* Jordan and Dickerson, 1908, and the new species *Bairdiella goeldi*.

It is important to note that the taxonomic reviews of both Marceniuk et al. [11] and Chao et al. [8] found considerable overlap in several morphological traits between species, which hampered the reliable identification of *Stellifer* and *Bairdiella* species. The molecular phylogeny of Lo et al. [7] also revealed a high level of genetic similarity between *Corvula macrops* Steindachner, 1875 and *Odontoscion xanthops* Gilbert, 1898, which led these authors to emphasize the need for a review of these genera. In addition, two lineages of *Odontoscion dentex* Cuvier, 1830, were identified in the Atlantic Ocean [12], which indicates the presence of undescribed cryptic diversity, given that *O*. *dentex* is the only valid species known to occur in the western Atlantic. All these findings further reinforce the need for a thorough assessment of the species-level diversity of the subfamily Stelliferinae.

The species is the fundamental taxonomic unit, and as such, the correct identification and delimitation of species are essential for reliable research in the fields of biodiversity, systematics, evolution, and ecology [13, 14]. However, the reliable identification of species is rarely straightforward, and the difficulties of defining a species based on empirical data have led to the development of increasingly sophisticated methods of analysis, known as species delimitation methods [15, 16].

Many of these delimitation approaches employ DNA markers, which have been shown to be an effective tool for the identification of species and have made a significant contribution to the taxonomy of several animal groups [15–22]. One of the genomic regions most used in the species delimitation approach is the DNA barcoding sequence, a fragment of approximately 650 base pairs (bps) of the cytochrome oxidase C subunit I (COI) gene, which is effective for the discrimination of metazoan species based on the comparison of their intra- and interspecific divergences [22–26].

Given the current lack of consensus on the species diversity of the stelliferine and the increasing application of molecular analyses for the elucidation of taxonomic questions, the present study was based on the application of species delimitation methods using the DNA barcoding marker to assess the diversity and delimit the interspecific barriers within the Stelliferinae. Also, in cases of cryptic lineages, we evaluate phylogeographic patterns to infer potential barriers to gene flow that may have influenced lineages differentiation. In addition to their taxonomic significance, the results of this research provide valuable insights for the development of effective public policy for the conservation of this important fishery resource.

## Materials and methods

### Ethics statement

There were no endangered or protected species between the samples used in the present study. In Brazil, the samples were purchased from artisanal fisherman, and permission to undertake collection, handling, transportation, and DNA extraction was obtained in the name of Dr. Simoni Santos by the Brazilian Environment Ministry (Permit number 18401–3). The samples of Costa Rica were collected under permissions of the Sistema Nacional de Áreas de Conservación (Permit number R-SINAC-SE-DT-PI-003-2021) and the Comisión Nacional para la Gestión de la Biodiversidad (Permit number R056-2015-OT-CONAGEBIO), accessed through Resolución No. 377 of the Vicerectoría de Investigación of the University of Costa Rica (UCR) and are deposited in the Fish Collection of the Zoology Museum of the UCR. The samples from Mexico were collected under permits PPF/ DGOPA-035/15 and CONAPESCA-PPF/DGOPA-262/17, and fishes from Ecuador were collected under permit 013/2012 PNG/N21-2017-EXP-CM-2016-DNB/MA. The samples from Mexico and Ecuador are deposited in the Fish Collection of the Universidad Michoacana de San Nicolás de Hidalgo, Mexico.

For the samples collected in Brazil, approval by the ethics committee was not requested because the fish were purchased from artisanal fishermen and were already dead at the time of collection.

### Sampling

The present study was based on the analysis of 160 samples, including 57 specimens accessed during the present study from the western Atlantic and eastern Pacific oceans, while all the others were obtained from GenBank (Table 1). The samples included all stelliferine genera and 31 valid species, the equivalent of 60.8% of the recognized taxa of this subfamily.

**Table 1:** Stelliferinae species analyzed in the present study.

Species	n	Origin	GenBank accession number
*Stellifer punctatissimus*	10	Western Atlantic	KJ907238, KJ907239, MG494841–MG494845, MG494885–MG494887
*Stellifer menezesi*	4	Western Atlantic	OQ872161–OQ872164*
*Stellifer gomezi*	3	Western Atlantic	OQ872165–OQ872167*
*Stellifer scierus*	5	Eastern Pacific	MG494899–MG494902, OQ872168*
*Stellifer strabo*	5	Eastern Pacific	MG494903–MG494905, OQ872169*, OQ872170*
*Stellifer typicus*	5	Eastern Pacific	MG494906–MG494909, OQ872171*
*Stellifer simulus*	5	Eastern Pacific	MG494912–MG494915, OQ872172*
*Stellifer vermicularis*	5	Eastern Pacific	OQ872173–OQ872177*
*Stellifer microps*	3	Western Atlantic	KJ907246, KP722779, KJ907247
*Stellifer brasiliensis*	5	Western Atlantic	KJ907243–KJ907245, OQ872178*, OQ872179*
*Stellifer stellifer*	5	Western Atlantic	JQ365589, JQ365590, MT879850, KJ907264, KJ907261
*Stellifer rastrifer*	5	Western Atlantic	KJ907251–KJ907255
*Stellifer naso*	5	Western Atlantic	MG494916, MG494917, KJ907249, KJ907250, OQ872180*
*Stellifer mancorensis*	5	Eastern Pacific	OQ872181–OQ872185*
*Stellifer ericymba*	4	Eastern Pacific	KP722778, OQ872186–OQ872188*
*Stellifer oscitans*	2	Eastern Pacific	KP722780, OQ872189*
*Stellifer illecebrosus*	2	Eastern Pacific	OQ872190*, OQ872191*
*Stellifer minor*	5	Southwestern Pacific	KY572896, KY572897, KY572902–KY572904
*Stellifer lanceolatus*	5	Western Atlantic	MT456230, MT456115, MT455504, MT455276, MT455122
*Stellifer chrysoleuca*	1	Eastern Pacific	MT879847
*Stellifer collettei*	6	Western Atlantic	JX124903, KJ907256–KJ907260
*Bairdiella ronchus*	3	Western Atlantic	KJ907231, KJ907232, MG813778
*Bairdiella goeldi*	8	Western Atlantic	MG820457, MG820456, MG813775–MG813777, OQ872192–OQ872194*
*Bairdiella armata*	5	Eastern Pacific	OQ872195–OQ872199*
*Bairdiella chrysoura*	10	Western Atlantic	MT455937, MT455546, MT455182, MT455162, MT455034, GU225144–GU225148
*Bairdiella veraecrucis*	1	Western Atlantic	MG813774
*Odontoscion dentex*	20	Western Atlantic	KJ907233, KJ907234, JQ842974, JQ842975, JQ841298–JQ841302, MF999167–MF999170, HM389625, OQ872200–OQ872205*
*Odontoscion xanthops*	2	Eastern Pacific	KP722748, OQ872206*
*Corvula macrops*	10	Eastern Pacific	KP722711, KP72212, OQ872207–OQ872214*
*Corvula sanctaeluciae*	3	Western Atlantic	MG813779–MG813781
*Elattarchus archidium*	3	Eastern Pacific	OQ872215–OQ872217*

n = number of individuals used in the analyses, including samples accessed in present study (*) and those obtained from GenBank.

The stelliferine taxa collected for this study were identified based on their morphology using taxonomic keys [8, 27, 28], and the taxonomic identity of the sequences obtained from GenBank were maintained according to their original submission. Samples of muscle tissue or pectoral fin of each individual were taken and stored in 90% ethanol prior to processing in the laboratory.

### DNA extraction, PCR, and sequencing

Total DNA was extracted from the tissues using the Wizard Genomic DNA Purification kit (Promega) following the manufacturer’s protocol. The concentration and purity of the DNA were evaluated in a Nanodrop 2000 (Thermo Scientific), and whenever necessary, the samples were electrophoresed in a 1% agarose gel stained with GelRed to evaluate the quality of the DNA under an ultraviolet transilluminator.

The COI barcoding region was amplified by Polymerase Chain Reaction (PCR), using the primers FishF1 and FishR1 [29]. The PCRs were run in a final volume of 15 μl, containing 2.4 μl of dNTPs (1.25 mM), 1.5 μl of 10 X buffer solution, 0.5 μl of MgCl_2_ (50 mM), 0.3 μl of each primer (10 pmol/μl), 1–3 μl of genomic DNA (100 ng/μl), 0.12 μl of Taq DNA polymerase (5 U/μl), and water to complete the final reaction volume. The cycling conditions were based on the protocol described by Silva et al. [10].

The PCR products were purified using the polyethylene glycol-8000 M protocol [30], and the sequencing reaction was based on the dideoxyterminal method [31] using the Big Dye 3.1 terminator kit (Applied Biosystems) following the manufacturer’s instructions. The samples were sequenced in an ABI 3500XL Genetic Analyzer (Applied Biosystems, Inc.).

### Data analysis

The sequences were aligned automatically using CLUSTAL W [32], which was run in BioEdit 5.0.6 [33]. The sequences were corrected manually whenever necessary.

PartitionFinder 2 software [34] was used to select the best partition layout and the model of molecular evolution to be used for the Maximum Likelihood (ML) analyses, run in RAxML 8.1.5 [35], and the Bayesian inference (BI), run in BEAST 2.5 [36]. The ML and BI trees were used as input files for species delimitations analysis in bPTP and GMYC, respectively.

The species were delimited using three single locus analysis methods: (i) Automatic Barcode Gap Discovery—ABGD [17], (ii) Generalized Mixed Yule Coalescence—GYMC [18, 19], and (iii) Bayesian Poisson Tree Process—bPTP [20].

For barcoding gap inference was used the ABGD program, available at <https://bioinfo.mnhn.fr/abi/public/abgd/>. The input for this analysis was provided by a pairwise distance matrix obtained from MEGA 11 [37] using the Kimura 2-Parameter model [38] with the following parameters: Pmin = 0.001 and Pmax = 0.1, steps = 10, Nbins = 20, and X (relative gap width) = 1.5, Jukes-Cantor JC69 model, as proposed by the ABGD.

The GMYC analysis was performed on the online platform at <https://species.h-its.org/gmyc/>, using the single threshold method and with the input being provided by an ultrametric Bayesian guide tree obtained from BEAST 2.5. Two independent runs were conducted based on 10 million Markov Chain Monte Carlo (MCMC) generations, which were sampled every 1000 generations, with 10% burn-in. The Yule speciation process was assumed with a relaxed uncorrelated lognormal clock and GTR evolutionary model, with all the other parameters being set at the default values. The run parameters were verified over the course of the generations, and the convergence of the data was evaluated in Tracer 1.7.1 [39]. Only the runs with an effective sample size (ESS) of over 200 were considered in the analyses. The TreeAnnotator 2.7.5 implemented in the BEAST 2.5 [36] was used to construct a consensus tree.

The bPTP analysis was run on the online platform <http://species.h-its.org/ptp/>, with a Maximum Likelihood (ML) guide tree being used as the input file. The ML tree was generated in RAxML 8.1.5 [35] based on the GTR model with a shotgun search, which allows for variation in the branch lengths of the tree. The statistical support for the branches was estimated using 1000 bootstrap pseudoreplicates. The parameters applied for the implementation of the ML tree on the online bPTP platform were based on 100,000 MCMC generations, with the recommendations of the program being considered for fewer than 50 taxa, that is, a standard thinning of 100 and 10% burn-in and, as suggested by Zhang et al. [20], the convergence of the runs was verified.

The sequence database employed for the delimitation of the species was also used to generate a matrix of genetic distance (K2P) in MEGA 11 [37], which was the basis for the evaluation of the intra- and interspecific divergence of the stelliferine taxa. In this analysis, the GenBank sequences KJ907231 and KJ907232, identified as *B*. *ronchus*, were considered to be *B*. *goeldi*, as proposed by Marceniuk et al. [11]. In addition, the divergence between GenBank sequence KP722779, deposited as *Stellifer microps* Steindachner, 1864, was compared not only with the other *S*. *microps* sequences but also with those of *Stellifer brasiliensis* Schultz, 1945, given that this particular sequence was aligned with the *S*. *brasiliensis* group in all the species delimitation analyses. The distinct lineages identified by the delimitation methods in *Bairdiella chrysoura* Lacepède, 1802 and *O*. *dentex* were considered to be different groups for the analysis of the intra- and interspecific distances.

Finally, a total of 20 sequences of *O*. *dentex* and 32 sequences of *B*. *chrysoura* were used to construct haplotype networks in Haploviewer [40], using an ML tree as the guide. These networks were generated for the evaluation of the genealogy and geographic distribution of the haplotypes of the *O*. *dentex* and *B*. *chrysoura* lineages.

## Results

### Species delimitation

The species delimitation analyses were based on a 584 bp fragment of the COI barcoding region sequenced in the 160 specimens, which represent 31 nominal species belonging to all five currently recognized Stelliferinae genera (*Stellifer*, *Bairdiella*, *Odontoscion*, *Corvula*, and *Elattarchus*).

**Fig 1:**
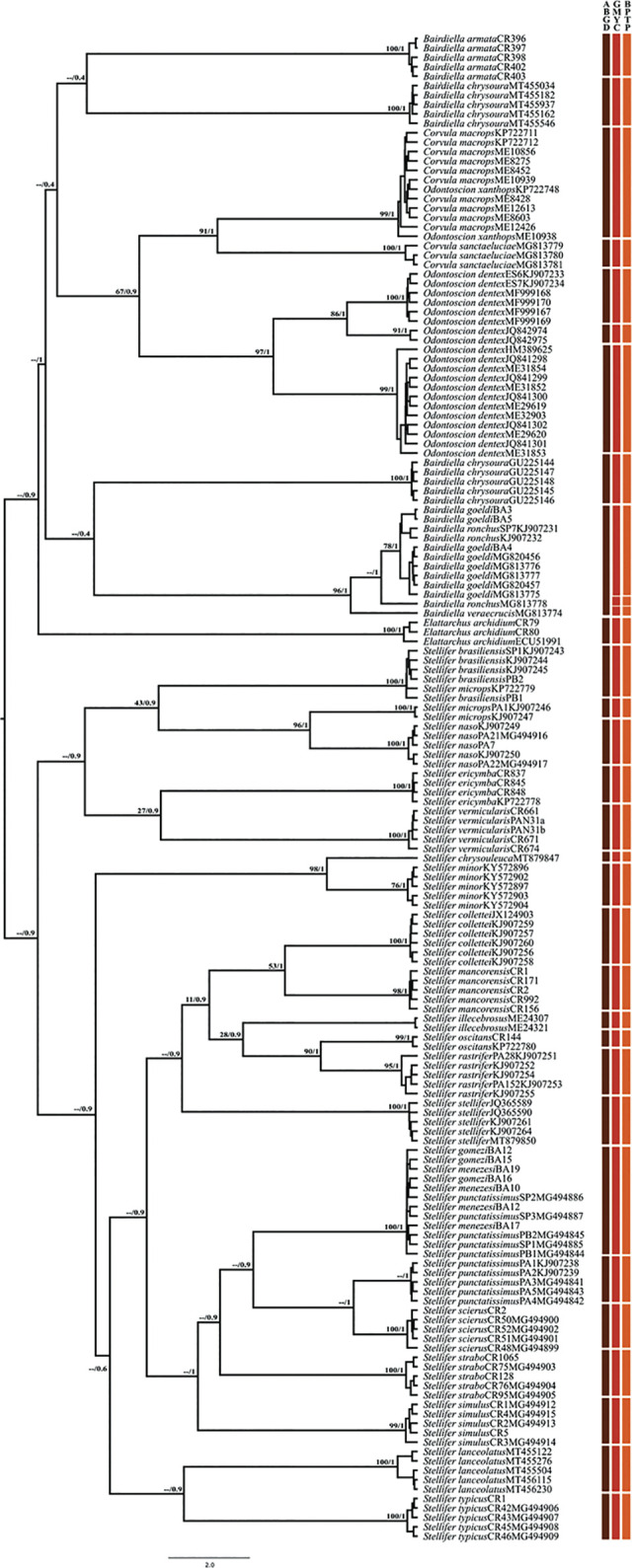
Bayesian inference tree of Stelliferinae based on COI barcoding region. Numbers above branches indicate posterior probability of respective grouping. Vertical bars represent ABGD, GMYC, and bPTP species delimitation methods.

The ABGD analysis returned nine partitions consisting of 27–58 molecular operational taxonomic units (MOTUs), of which partition 6 (maximum prior distance, p = 0.0129), which had 30 MOTUs, was the most congruent with the data. The GMYC analysis recovered 32 MOTUs (with a confidence interval of 32–33, without the inclusion of an outgroup; maximum likelihood of the null model = 579.1894; maximum likelihood of the GMYC model = 642.9621; threshold time = −0.4879149). The bPTP analysis also delimited 32 MOTUs. The disagreement on the number of MOTUs delimited by the three methods is explained by the fact that the ABGD allocated *B*. *ronchus*, *B*. *goeldi*, and *B*. *veraecrucis* in the same MOTU, while they were differentiated in the bPTP and GMYC approaches (Fig 1).

In general, the tests were congruent in their attribution of the valid stelliferine species to distinct MOTUs. In the genus *Stellifer*, however, one of the specimens of *S*. *microps* (KP722779) was grouped with the *S*. *brasiliensis* cluster, while *S*. *menezesi* and *Stellifer gomezi* Cervigón, 2011 were included in the same MOTU (Fig 1). In the case of the genus *Corvula*, in addition, all three methods delimited *Corvula sanctaeluciae* Jordan, 1980 as a distinct lineage, whereas *Corvula macrops* was grouped with *O*. *xanthops* (Fig 1).

**Fig 2:**
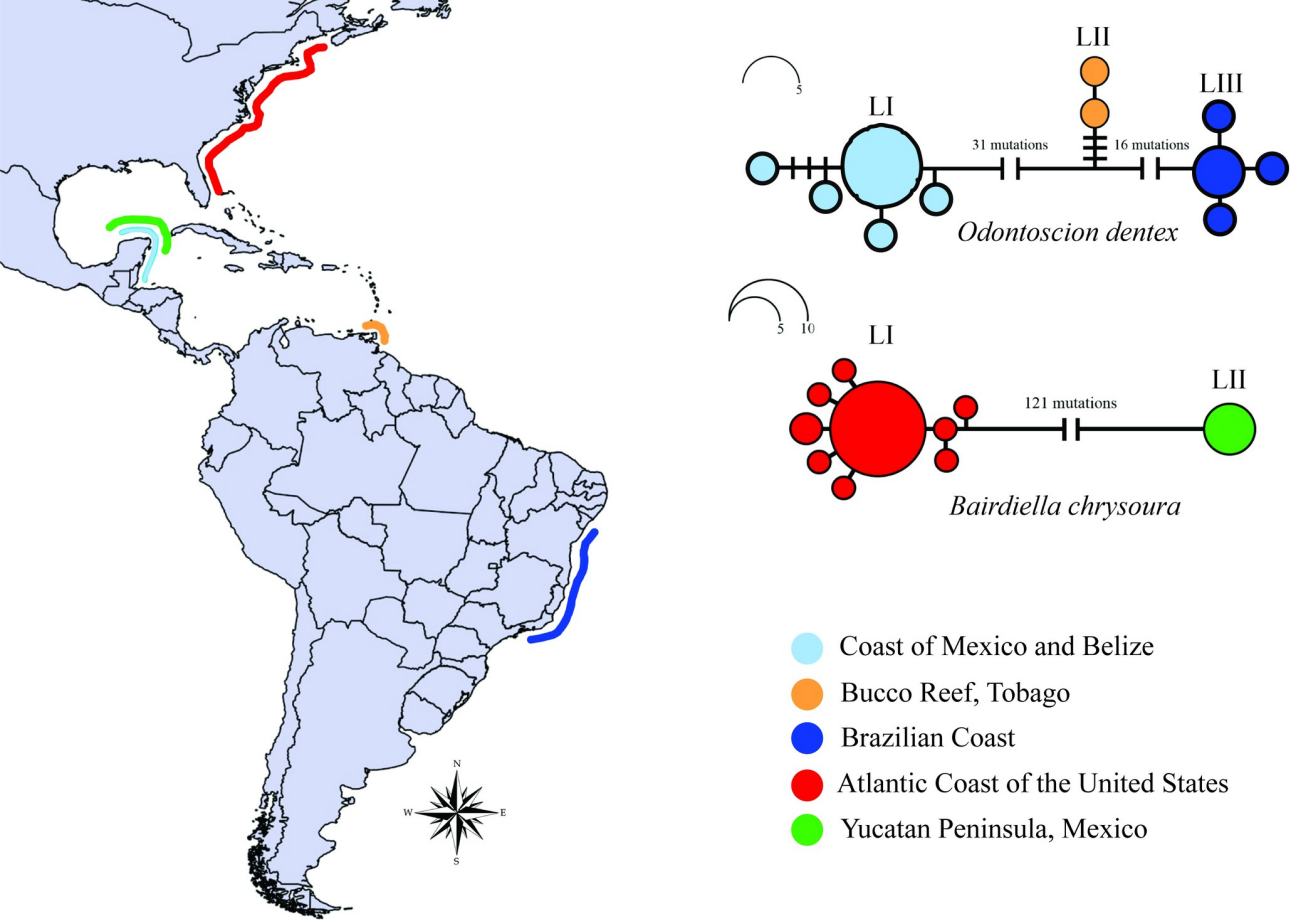
Distribution map of *Odontoscion dentex* and *Bairdiella chrysoura* lineages and haplotype network based on maximum likelihood tree from COI region.

All three methods also allocated *Elattarchus archidium* Jordan and Gilbert, 1882 to a single lineage, which is consistent with the monotypic status of the genus (Fig 1). In the genus *Odontoscion*, by contrast, all three methods returned three lineages for *O*. *dentex*, each with a disjunct geographic distribution–(i) one lineage found in the coastal regions of Mexico and Belize, (ii) one from the island of Tobago, and (iii) a third lineage composed of individuals from the Brazilian coast (Figs 1 and 2).

While all the individuals of *Bairdiella armata* Gill, 1863 were allocated to a single MOTU (Fig 1), the different approaches disagreed on the arrangement of *B*. *ronchus*, *B*. *goeldi*, and *B*. *veraecrucis*, which were grouped in the same MOTU in the ABGD but in distinct groups in the bPTP and GMYC analyses (Fig 1). In the case of *B*. *chrysoura*, two highly divergent lineages were delimited, which had disjunct distributions, one containing specimens from the Atlantic coast of the United States and the other from the Gulf of Mexico (Figs 1 and 2).

### Genetic divergence and genealogy of the haplotypes

The analysis of intra- and interspecific divergence was based on the individuals in the database used for the species delimitation analyses, although Table 2 shows only the divergence between taxa for which the results of the delimitation analyses disagreed with the valid species. The mean intraspecific divergence ranged from 0.0% to 0.52%, which was consistent with the allocation of the individuals to the different groups in the delimitation tests (Table 2).

**Table 2:** Mean pairwise intra- and interspecific genetic distances (K2P) recorded in present study between Stelliferinae taxa.

	Taxon	1	2	3	4	5	6	7	8	9	10	11	12	13	14	15	16
**1**	*Stellifer punctatissimus*	**0**															
**2**	*Stellifer menezesi*	0.111	**0**														
**3**	*Stellifer gomezi*	0.111	0	**0**													
**4**	*Stellifer microps*	0.181	0.180	0.180	**0**												
**5**	*Stellifer brasiliensis*	0.162	0.165	0.165	0.134	**0.002**											
**6**	*Bairdiella ronchus*	0.169	0.151	0.151	0.183	0.162	**n/c**										
**7**	*Bairdiella goeldi*	0.169	0.158	0.158	0.186	0.159	0.020	**0.005**									
**8**	*Bairdiella veraecrucis*	0.170	0.151	0.151	0.174	0.162	0.030	0.040	**n/c**								
**9**	*Bairdiella armata*	0.189	0.204	0.204	0.171	0.176	0.167	0.170	0.167	**0.001**							
**10**	*Bairdiella chrysoura*I	0.178	0.195	0.194	0.160	0.176	0.189	0.187	0.184	0.191	**0.001**						
**11**	*Bairdiella chrysoura*II	0.210	0.213	0.213	0.187	0.187	0.205	0.204	0.198	0.200	0.237	**0**					
**12**	*Odontoscion dentex*III	0.188	0.192	0.192	0.163	0.166	0.164	0.172	0.166	0.200	0.178	0.204	**0.002**				
**13**	*Odontoscion dentex*I	0.198	0.192	0.192	0.164	0.157	0.174	0.180	0.177	0.189	0.180	0.200	0.093	**0.003**			
**14**	*Odontoscion dentex*II	0.185	0.177	0.176	0.157	0.160	0.153	0.161	0.156	0.179	0.173	0.190	0.040	0.072	**0.002**		
**15**	*Odontoscion xanthops*	0.168	0.183	0.183	0.174	0.178	0.168	0.174	0.173	0.191	0.169	0.214	0.149	0.144	0.130	**0.005**	
**16**	*Corvula macrops*	0.168	0.182	0.182	0.174	0.175	0.168	0.172	0.170	0.188	0.169	0.215	0.151	0.147	0.132	0.004	**0.002**

Values in bold represent mean intraspecific genetic distance; n/c = not calculated.

In the genus *Stellifer*, the divergence values ranged from 0.02% between *S*. *menezesi* and *S*. *gomezi* to 23.26% between *Stellifer ericymba* Jordan and Gilbert, 1882 and *S*. *punctatissimus* (Table 2). The GenBank sequence KP722779, which was identified as *S*. *microps*, was attributed in the present study to *S*. *brasiliensis* in all the delimitation tests and presented a high level of genetic similarity with this taxon (99.8%), in contrast with a mean divergence of 0.9% between this individual and the other specimens identified as *S*. *microps*.

A mean genetic divergence of 12.97% was recorded in the genus *Bairdiella*, ranging from 2.02% between *B*. *ronchus* and *B*. *goeldi* to 23.72% between the *B*. *chrysoura* lineages. These findings are consistent with those of the species delimitation analyses (Table 2). The mean divergence between the *Odontoscion dentex* lineages (Table 2) varied from 3.95% (lineage II *vs*. lineage III) to 9.33% (lineage I *vs*. lineage III). Moreover, a low level of divergence (0.4%) was found between *O*. *xanthops* and *C*. *macrops* (Table 2), which is consistent with the results of the species delimitation analyses that allocated the two species to the same MOTU (Fig 1).

The haplotype networks further reinforce the high level of divergence between the lineages of both *B*. *chrysoura* (I and II) and *O*. *dentex* (I, II and III). Moreover, *Bairdiella chrysoura* lineages I and II were separated by 121 mutations (Fig 2), while those of *O*. *dentex* were differentiated by at least 16 mutations (Fig 2).

## Discussion

### Species delimitation

This is the first study that has applied species delimitation methods to the assessment of the diversity and delimit the interspecific barriers within the subfamily Stelliferinae. Just over 60% of the valid species of this subfamily were sampled, representing taxa in both the western Atlantic and the eastern Pacific. In general, the three species delimitation methods were congruent in their allocation of valid species to distinct MOTUs, except in the case of *Bairdiella*, in which three taxa (*B*. *goeldi*, *B*. *ronchus*, and *B*. *veraecrucis*) were allocated to a single MOTU in the ABGD analysis.

In contrast, the three methods were in agreement on the assignment of the specimen of *S*. *microps* (KP722779) to *S*. *brasiliensis*, the allocation of *S*. *menesezi* and *S*. *gomezi* to the same MOTU, and the differentiation of lineages within both *O*. *dentex* and *B*. *chrysoura*, as well as the allocation of *O*. *xanthops* and *C*. *macrops* to a single MOTU. Therefore, while a single locus delimitation analysis does have limitations in detecting incomplete lineage sorting, recent hybridization or speciation [21, 41] and possible incongruities between the gene and species trees [42], the congruence of the results of the different approaches, which are based on distinct analytical assumptions and theoretical premises, are indicative of the robustness of the delimited MOTUS. In this case, the findings of the present study indicate that Stelliferinae has undescribed diversity, and that the taxonomy of the subfamily should be revised.

All three species delimitation methods grouped *S*. *menezesi* and *S*. *gomezi* in a single MOTU, which contradicts the results of Chao et al. [8], who described *S*. *menezesi* as a new species, distinguishing it from *S*. *gomezi*. These authors found morphological differences between the two species, including the height of the body, the length of the snout, and the size and shape of the nostrils, as well as the length of the pectoral fin and the second spine of the anal fin. However, these species overlapped in most of the morphological and meristic traits evaluated by Chao et al. [8], and the analyses presented here found a level of genetic divergence between the two taxa of 0.02%, which is typical of intraspecific divergence in the DNA barcode [29]. Therefore, it is possible that *S*. *gomezi* and *S*. *menezesi* represent a single species, as indicated by the species delimitation analyses. On the other hand, if these are in fact distinct species, it is possible that the pattern observed here is the result of incomplete lineage sorting or introgression, although it would only be possible to confirm this through a more comprehensive analysis based on additional loci, such as nuclear markers, and a more ample sample to better evaluate the differentiation of the taxa.

All the species delimitation methods allocated *S*. *microps* (KP722779) to the *S*. *brasiliensis* group, and this sequence was genetically more similar to those belonging to *S*. *brasiliensis* than it was to any of the other *S*. *microps* sequences. This indicates a possible error in the identification of the specimen deposited in GenBank, which is not uncommon and may occur due to either the morphological similarities of the species or errors during data submission [43, 44]. On the other hand, this situation may also be related to specific evolutionary processes, such as introgression or incomplete lineage sorting, which can only be resolved by the analysis of nuclear markers and a more comprehensive sampling of the different taxa involved. Therefore, a taxonomic revision must be performed concerning these species, including the analysis of morphological and molecular data and encompass the accurate identification of vouchers utilized in previous molecular investigations.

In the case of the genus *Bairdiella*, there was disagreement between the ABGD and the other two methods on the arrangement of *B*. *ronchus*, *B*. *goeldi*, and *B*. *veraecrucis*. The genetic divergence between these taxa, between 2% and 4% (Table 2), is consistent with species-level differentiation in DNA barcoding. Marceniuk et al. [11] used morphological data and DNA barcoding to revise the genus *Bairdiella* from the western South Atlantic and concluded that this region is occupied by a species complex formed by *B*. *ronchus*, *B*. *goeldi*, and *B*. *veraecrucis*, which is consistent with the results of coalescence-based methods (bPTP and GMYC) and the genetic divergence recorded here. In his review of *Bairdiella*, Marceniuk et al. [11] suggested that *B*. *ronchus* is restricted to the Caribbean region, while *B*. *goeldi* occurs on the Brazilian coast, thus specimens deposited in GenBank as *B*. *ronchus* (KJ907231 and KJ907232) were reclassified by these authors as *B*. *goeldi* and in our analyzes they grouped in the MOTU referring to this taxon, corroborating that proposal. Therefore, our results do not reflect a case of *B*. *ronchus* paraphyly, but they do reveal the need to update the taxonomy of *B*. *ronchus* specimens (KJ907231 and KJ907232) in GenBank.

The species *Corvula macrops* and *Odontoscion xanthops*, which occur in the eastern Pacific, were allocated to the same MOTU in all the analyses and had a low level of genetic divergence (0.4%), which indicates that they belong to the same taxon. All previous phylogenetic analyses that included *Corvula* and *Odontoscion* have found that these two genera are closely related [5, 7, 10]. In a multilocus analysis, Lo et al. [7] also identified a high level of genetic similarity between *C*. *macrops* and *O*. *xanthops* and concluded that the presence of canines in *Corvula* as a diagnostic trait for the differentiation of the two genera should be re-evaluated. In fact, the morphological similarities between these two genera led to the allocation of some *Corvula* species to *Odontoscion* [45]. This suggests that these two taxa belong to the same genus and that *C*. *macrops* and *O*. *xanthops* should be a single species, although more detailed analyses, including all species currently described in both genera and using both morphological and molecular data, would be necessary to corroborate this conclusion.

### Phylogeographic patterns and taxonomic implications in cryptic lineages of Stelliferinae

The species delimitation analyses confirmed the presence of two *B*. *chrysoura* lineages in the western Atlantic, supported by their high level of genetic divergence (23.7%) and their clear differentiation in the haplotype network. Within *Bairdiella*, only *B*. *chrysoura* occurs in the North Atlantic, which could indicate that the differentiation observed in the present study is not due to identification errors. The two lineages identified in the present study had disjunct distributions, with lineage I occurring in the North Atlantic (United States) and lineage II in the Gulf of Mexico, off the Yucatan Peninsula in Mexico. The Florida Peninsula is known to have a profound influence on the circulation patterns of the local marine currents and forms a barrier between the coastal region of the North Atlantic and the Gulf of Mexico, restricting dispersal between the populations of different species found on the two sides of the peninsula, which limits gene flow and supports the differentiation of many taxa [46–48], including those of the sciaenids [49–51]. Given these considerations, the two lineages are very likely distinct species, although further research is needed for the validation of the taxa.

All three species delimitation methods were in agreement on the allocation of the *O*. *dentex* specimens to three different MOTUs, as confirmed by the haplotype network and the genetic divergence of 4.0% to 9.3% between the lineages. These findings are consistent with the hypothesis of cryptic speciation within *Odontoscion* proposed by Duarte et al. [12], who, using DNA barcoding, identified two groups with a divergence of 12.9% between the Caribbean and Brazilian provinces. Lineages I and II of *O*. *dentex* occur in the Caribbean, where they inhabit areas influenced by distinct marine currents. There are two well-defined biogeographic barriers that differentiate the fish populations found in this area, one in the eastern Caribbean and the other on the northern edge of the Nicaraguan Rise [52], which may be directly responsible for the differentiation of the two lineages. *Odontoscion dentex* is a reef fish, and although the tempo and mode of its larval dispersal are not known, studies of other reef-dwelling fish indicate that the behavior of the larvae, in particular their habitat use, combined with the local marine currents, influence the connectivity of the populations in the Caribbean [53–55]. In addition to these processes in the Caribbean, the differentiation of these lineages from that in Brazil (lineage III) may also be related to the freshwater plume formed by the discharge of the Amazon and Orinoco rivers, which flows into the Atlantic Ocean and is known to limit gene flow and contribute to the diversification of the reef-dwelling taxa of the Brazilian and Caribbean provinces [56–58].

## Conclusions

The species delimitation approach proved effective for the diagnosis of the majority of valid stelliferine species and their allocation to distinct MOTUs, which was consistent, in most cases, with their current taxonomic arrangement [3, 4]. While a single locus approach has certain limitations for the delimitation of species and multilocus methods would be preferable for the inference of interspecific limits [59, 60], species delimitation based on DNA barcoding is an important initial approach for the understanding of the diversity of taxa in poorly studied groups, as is the case of Stelliferinae. All three methods were congruent on the question of the undescribed diversity in *B*. *chrysoura* and *O*. *dentex*, as well as the clear need for a taxonomic review of *S*. *menezesi*, *S*. *gomezi*, *Corvula*, and *Odontoscion*. Clearly, while the results of the present study offer important insights into the alpha taxonomy of the stelliferines, they also reinforce the need for a more detailed review of a number of taxa. More extensive studies, which include most or, preferably, all of the taxa of the subfamily and an integrative taxonomic approach based on multilocus or genomic and morphological data, would provide a more conclusive interpretation of the taxonomy of the subfamily. The findings of these studies will also be fundamental for the development of more detailed ecological, phylogenetic, and phylogeographic research, as well as the formulation of effective conservation strategies for stelliferine species.
